# Gallbladder perforation: a rare complication of postoperative chemotherapy of gastric cancer

**DOI:** 10.1186/s12957-015-0659-6

**Published:** 2015-08-15

**Authors:** Yanlai Sun, Wentao Song, Qingsheng Hou, Jianning Li, Hongliang Guo

**Affiliations:** Department of Colorectal Cancer Surgery, Shandong Cancer Hospital and Institute, 440 Jiyan Road, Jinan, 250117 China; Department of Anesthesiology and Operation, Affiliated Hospital of Shandong Academy of Medical Sciences, 38 Wuyingshan Road, Jinan, 250031 China

**Keywords:** Gallbladder perforation, Postoperative chemotherapy, Gastric cancer, Complication

## Abstract

A middle-aged man presented 1 day after being discharged from hospital with completing the first course of postoperative chemotherapy. He suffered a sudden persistent high fever and chills. It was noted that he had a history of a total gastrectomy (with D2 lymphadenectomy) 1 month ago. His admission bloods revealed total bilirubin was 142.2umol/L , indirect bilirubin of 107.6umol/L and white cell count of 20.05×10^9^/L. A color doppler ultrasound scan confirmed fluid and gas around liver and hilus lienis while the gallbladder cannot be detected. During Computed Tomography (CT) guided puncture positioning technology and setting a three-channel tube, about 400 ml of foul smell hazel turbid liquid was drained out. He was diagnosed as gallbladder perforation and he was underwent conservative treatment consist of drainage, banning diet, total parenteral nutrition and intravenous antibiotics. Then he recovered well within the subsequent 10 days and was discharged.

## Background

Perforation of the gallbladder is a rare but life-threatening condition, which usually requires immediate surgical intervention [1, 2]. Sometimes perforation of the gallbladder may not be different from uncomplicated acute cholecystitis with high morbidity and mortality rates because of delay in diagnosis [3–4]. Perforation of the gallbladder is an extremely rare but serious complication of postoperative chemotherapy although the progress may mainly facilitated by operation [5–7]. There are no reported cases of these in the literature. There are no similar reported cases in the literature to the best our knowledge. In this article, we describe a case of a perforation of the gallbladder in a patient most likely related to a combination of operation and chemotherapy, which has not been reported yet.

## Case presentation

A 48-year-old man was presented to hospital because of persistent high fever and chills on the first day after completing the first course of chemotherapy. He has a history of a total gastrectomy with D2 lymphadenectomy 1 months ago and one course of postoperative chemotherapy which consisted of Oxaliplatin and S-1(Oxaliplatin 150mg ivdrip d1, S-1 po 40mg bid Day1-14) because of gastric cancer. He has no liver or biliary disease history.

At the time of admission, his temperature was up to 40 degree and blood pressure was 120/80mmHg. The heart rate and respiratory rate were 86/min and 22/min, respectively. The breath sound of lungs was clear. The whole skin appeared mild jaundice and sclera moderate yellow dye. There was no enlargement phenomenon of superficial lymph nodes. Abdominal examination revealed no pain or tightness. The liver and spleen was unpalpable. Murphy syndrome was negative. Other examination was unremarkable.

### Investigations

Baseline investigations revealed a aspertate aminotransferase of 97U/L, and the total bilirubin was 142.2umol/L, indirect bilirubin of 107.6umol/L, white cell count of 20.05×10^9^/L. His hepatitis virus examination was negative and renal function, Electrolyte was normal.

### Differential diagnosis

**Fig. 1:**
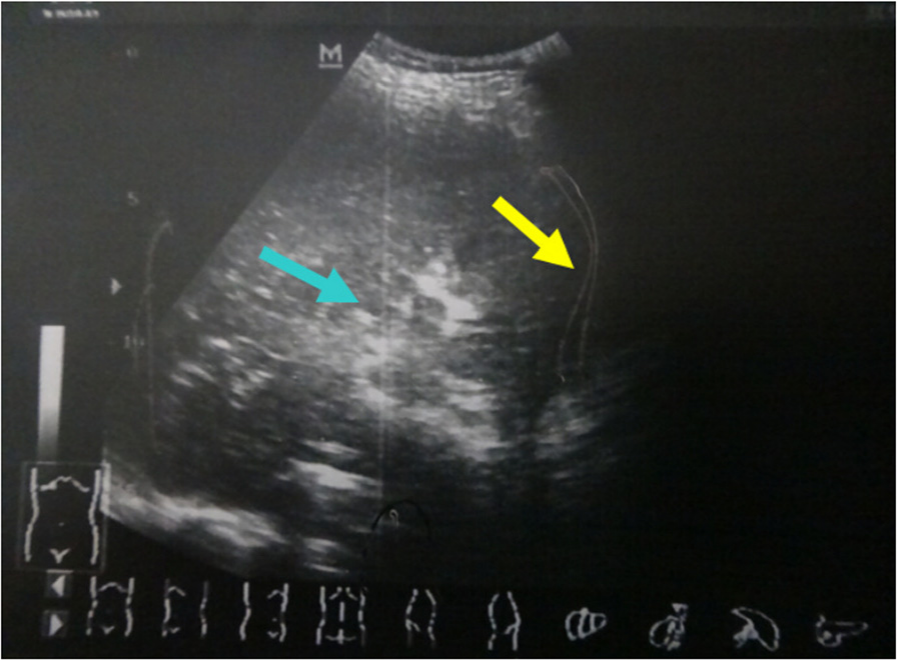
Color doppler ultrasound investigation. The ultrasound showing fluid around liver and hilus lienis (yellow arrow) and uneven right liver lobe (blue arrow). The gallbladder cannot be detected

An urgent color doppler ultrasound of his upper abdominal was performed which demonstrated right upper abdominal structures disorders as evidenced by the strong echo gas. Parenchyma of Right liver lobe was uneven and the gallbladder cannot be detected. There was a small amount effusion around liver and hilus lienis (Fig. 1).

**Fig. 2:**
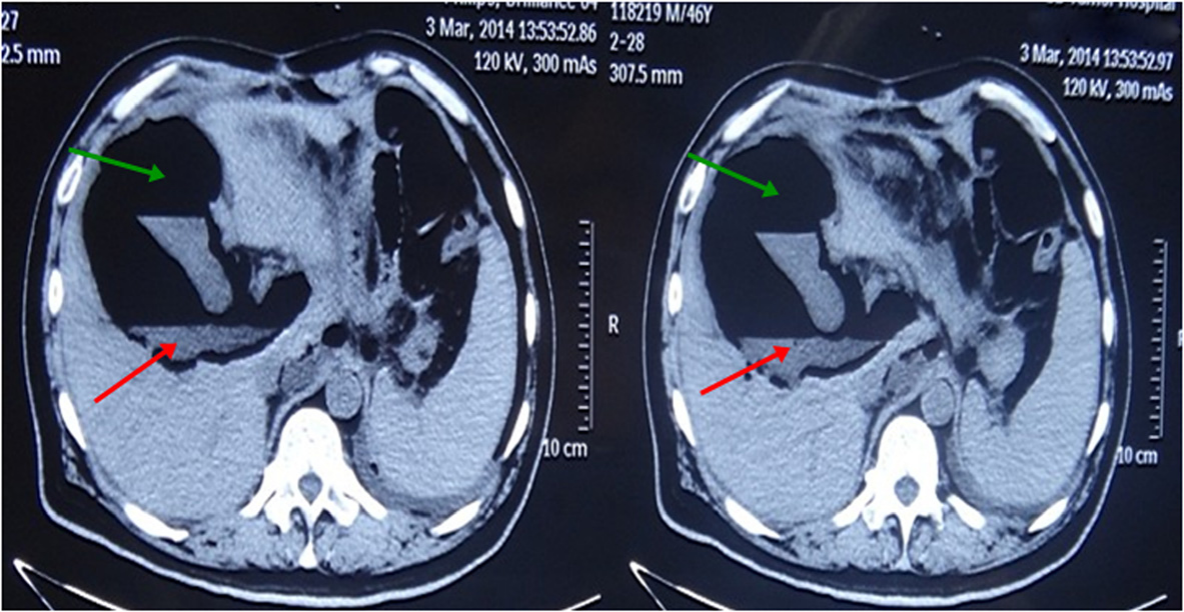
CT scans investigation. CT abdomen/pelvis showing fluid (red arrow) and gas (green arrow) in the liver, gallbladder cannot be detected

Both for confirm diagnosis and further treatment, he underwent an emergency CT guided puncture positioning technology and setting a three-channel tube (Fig. 2).Then about 400 ml of foul smell hazel turbid liquid was drained out.

### Treatment

Conservative treatment consist of banning diet, total parenteral nutrition and intravenous antibiotics was complemented at the time of admission while records the drainage quantity everyday.

### Outcome and follow-up

His temperature dropped to 37 degrees,and the jaundice was significantly improved by the first day of conservative treatment. His drainage quantity and liver function tests were noted to be persistently elevated (Table 1). However, blood picture normalised and drainage quantity gradually reduced over the next few days. He was switched to liquid diet by day 5 and continued to recover well. He was discharged 10 days with an outpatient appointment in 8 weeks.

**Table 1:** Progression of liver function tests and drainage quantity after conservative treatment

Liver function tests and drainage quantity	Day 1	Day 3	Day 7
total bilirubin(umol/L)	27.3	10.9	9.2
indirect bilirubin(umol/L)	11.9	5.1	4.5
Alkaline phosphatase(U/L)	113.5	76.1	32.7
Alanine transaminase(U/L)	261.7	116.0	49.9
drainage quantity(ml)	200	40	15

Review normal every 4 weeks and he had begun the second course of chemotherapy which consisted of oxaliplatin and fluorouracil (oxaliplatin 150mg ivdrip day1, 5-fluorouracil (5-FU) 500mg iv day1, 5-FU 2500mg civ day 2–3).

## Discussion

Perforation of the gallbladder is one of the rare but severe complications of acute calculous cholecystitis, like empyema, gallstone ileus, cholecystoenteric fistula and emphysematous cholecystits [8]. Acute acalculous cholecystitis is defied as acute cholecystitis without detection of any gallstones. Acute acalculous cholecystitis immediately after gastric operation is also rare [9]. Based on a Danish study, the risk of cholecystitis is 30 % higher in patients with cancer compared with general population [8]. But the data eventually develop to gallbladder perforation in total gastrectomy for cancer can’t be acquired. Perforation of the gallbladder can occur as early as 24 h after the onset of acute cholecystitis, or after a few days to weeks [10]. Ischemic changes of the gallbladder wall triggered by progression of local inflammation lead to perforation, which might explain why perforation occurs in the fundus, the most distant part from the main feeding artery, in more than half of cases [11]. Systemic vascular disorders, such as atherosclerotic cardiovascular disease and diabetes, immunosuppressed states, and malignancy are major risk factors for gallbladder perforation [12].

It is relatively rare that the gallbladder perforation developed without inflammation, cancer, foreign, stone, or body trauma which makes our study more unique and innovative. Uncommonly, gallbladder perforation may be related to the obstruction of bile duct or the destruction of the gallbladder wall, such as acute cholecystitis, gallstones, parasites, abdominal surgery, gallbladder vascular compression or obstruction. Chemotherapy can lead to gallbladder injury, but, to our knowledge there is no report of postoperative chemotherapy-induced gallbladder perforation.

In this case, there was no stone shown in the patients with color Doppler ultrasound examination. The following several surgical morbidity factors should be considered.

1. There is a study suggest that the biliary stasis is an important contributing factor in postoperative gallbladder complications [13]. Biliary stasis could cause the chronic cholecystitis and eventually leading to gallbladder perforation.
2. It has been reported that the risk of suffering from acute postoperative acalculous cholecystitis was 3.1 % higher in patient with radical gastrectomy compared with patient with simple gastrectomy [14].
3. A more suggestive cause of postoperative gallbladder perforation may be that the duodenum was excluded from the passage of large volumes of contents, resulting in possible stasis of bile and/or bacterial over growth within its lumen promoting bile inspissation, [15] which increased the pressure of gallbladder and then led to gallbladder inflammation and edema.
4. In this case, Gallbladder motility-related hormonal reduce, enteric nervous system function decline and pancreatic fistula may also play important roles in the gallbladder perforation.

It’s worth noting that the gallbladder perforation occured on the first day after completing the first course of chemotherapy. Chemo-therapic cholecystitis cases are not rare. In this gallbladder perforation case, chemotherapy-induced gall bladder injury is an important contributing factor.

In addition to the symptoms and signs, clinical picture and imaging is the first choice method for diagnosis of the disease. CT and ultrasonography are useful in making the diagnosis. Gallbladder wall thickening, peric-holecystic fluid collection, and a streaky omentum or mesentery is common findings of gallbladder perforation. Detection on imaging such as CT scans or color doppler ultrasound can be difficult in small perforations and the findings are usually non-specific that is, pericholecystic fluid/abscess and gallbladder cannot be detected. The presence of extra-luminal gallstones would be a suggestive sign, but it was not seen in this case [16]. In the event of a perforation, the first choice of treatment is urgent surgical intervention. However, conservative treatment which consisted of drainage, absolute diet, total parenteral nutrition and intravenous antibiotics could be given when the patient’s vital signs are stable and there are no symptoms of peritonitis. Avoiding unnecessary fasting and narcotics may be helpful to prevent the incidence of complication. While the presence of stones may increase the likelihood of an attack, preoperative studies undertaken routinely to discover their presence are not justified. A history of attacks of cholecystitis, particularly after previous operations, should be carefully sought.

## Conclusions

This case report is the first presentation of gallbladder perforation associated with operation and chemotherapy. The progression of gallbladder perforation may be mainly facilitated by operation, timing, scheme and dose should be more cautious when postoperative chemotherapy need to be implemented.

## Consent

Written informed consent was obtained from the patient for publication of this Case report and any accompanying images. A copy of the written consent is available for review by the Editor-in-Chief of this journal.

## Abbreviations

CT: Computed tomography
5-FU: 5-fluorouracil
